# circIPO7 dissociates caprin-1 from ribosomes and inhibits gastric cancer cell proliferation by suppressing EGFR and mTOR

**DOI:** 10.1038/s41388-023-02610-z

**Published:** 2023-02-03

**Authors:** Jing Liu, Liling Niu, Jiaru Hao, Yuan Yao, Meinan Yan, Hui Li

**Affiliations:** 1 Department of Gastrointestinal Cancer Biology, Tianjin Medical University Cancer Institute & Hospital, National Clinical Research Center for Cancer, Tianjin, 300060 China; 2 Key Laboratory of Cancer Immunology and Biotherapy, Tianjin, 300060 China

**Keywords:** Gastric cancer, Cell growth, Non-coding RNAs, Translation

## Abstract

Circular RNA (circRNA) is a novel RNA molecule characterized by covalently closed loop structure. Since its discovery, researchers have shown that circRNA is not “splicing noise” but a participant of various pathophysiological processes through unique mechanisms. circIPO7, which was identified as an independent prognostic factor in gastric cancer (GC) patients, was downregulated in GC tissues and cells compared to paracarcinoma tissues and normal epithelial cells. circIPO7 overexpression significantly suppressed GC cell proliferation in vitro and in vivo. Mechanistically, circIPO7 directly binds with caprin-1, an RNA-binding protein involved in mRNA translation, sharing overlapping binding sites with G3BP1. Thus, the complex containing overexpressed circIPO7 blocked the caprin-1-G3BP1 interaction and dissociated caprin-1 and its target mRNAs (EGFR and mTOR) from ribosomes, resulting in their translational inhibition, followed by PI3K/AKT/mTOR pathway inactivation. We uncovered a novel molecular mechanism for circRNAs in GC development, identifying circIPO7 as a potential target for cancer treatment.

## Introduction

Gastric cancer (GC) is one of the most common malignant tumors of the digestive system, with over one million new cases per year worldwide [1]. National guidelines recommend a multidisciplinary treatment strategy for patients combining surgical intervention, systemic chemotherapy, radiotherapy, immunotherapy and targeted therapy [2]. However, due to its difficulty of early detection, high heterogeneity and chemotherapy resistance, the overall survival of GC patients remains at 25% worldwide [3]. Therefore, more research is needed to elucidate the underlying molecular mechanisms and develop effective clinical biomarkers and therapeutic targets for GC.

Circular RNA (circRNA), a novel RNA molecule, has been found to be abnormally expressed in various cancers [4]. On the one hand, circRNAs are considered promising tumor biomarkers due to their high stability, conservation and tissue- and developmental specificity [5–7]. On the other hand, circRNAs can function as miRNA sponges, protein scaffolds, modulators of transcription, and translation templates [8–12]. The critical roles of circRNAs in diverse pathophysiological processes suggest their potential as therapeutic targets for GC. To date, research on circRNAs in GC is still in its infancy.

Cytoplasmic activation/proliferation-associated protein-1 (caprin-1), the prototype of a novel conserved family of RNA-binding proteins (RBPs) characterized by homology regions-1 and -2 (HR-1, HR-2), was originally identified in activated T lymphocytes [13]. HR-1 of caprin-1 is necessary for the formation of multimeric complexes, and RasGAP SH3-domain binding protein-1 (G3BP1) is the most prominent binding partner for caprin-1, which appears to provide a potential link between caprin-1 and cell proliferation [14]. Caprin-1 was reported to be aberrantly expressed in various cancers and associated with poor prognosis. Overexpression of caprin-1 in colon and ovarian cancer promoted cell proliferation and stemness respectively [15, 16]. In addition, the stress granules (SGs) assembled by caprin-1 and G3BP1 under stimulus conditions inhibited mRNA translation, thus enhancing cell survival and docetaxel resistance in prostate cancer [17]. However, the exact function and molecular mechanism of caprin-1 in GC are still unclear.

We previously reported that circIPO7 (circBase ID: hsa_circ_0005092) was positively associated with postoperative recurrence of GC [18]. Patients with lower circIPO7 expression may have shorter recurrence-free survival (RFS) and overall survival (OS). These results prompted us to explore the biological function of circIPO7 in GC development. In the present study, we demonstrate that the expression of circIPO7 is downregulated in GC. circIPO7 serves as an RBP sponge for caprin-1, inhibits the translation of downstream proliferation-related mRNAs, and ultimately suppresses malignant cell proliferation. Taken together, these results suggest that circIPO7 may be a potential therapeutic target for GC treatment.

## Results

### Identification and characterization of circIPO7 in GC

**Fig. 1:**
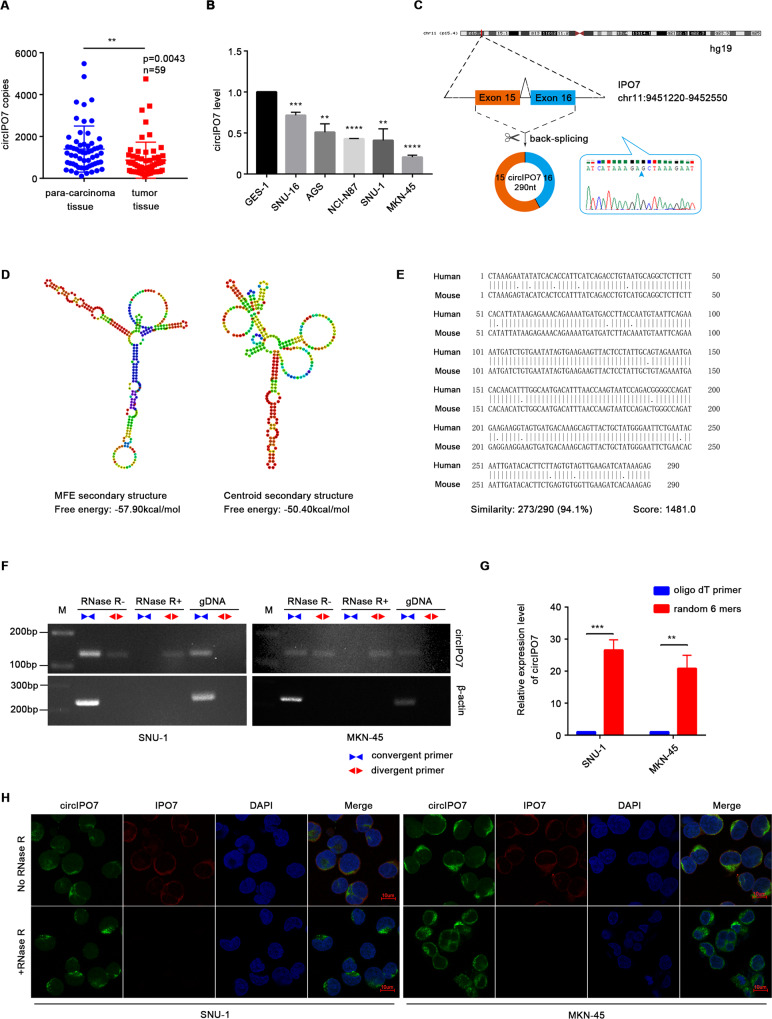
Identification and characterization of circIPO7 in GC. **A** The expression of circIPO7 in the tumor and paracancerous tissues of GC patients were examined by qRT-PCR. *n* = 59. **B** The expression of circIPO7 in gastric mucosal epithelial cell (GES-1) and GC cell lines (SNU-16, AGS, NCI-N87, SNU-1 and MKN-45) were examined by qRT-PCR. **C** Structures of the IPO7 genome and circIPO7 transcript. The circularization of circIPO7 was validated by Sanger sequencing. **D** The secondary structure of circIPO7 and their free energy was predicted by RNAfold. **E** Pairwise alignment of the human circIPO7 and mouse mmu-Ipo7_0007 sequences. **F** PCR validation of the circIPO7 amplified by divergent primers and convergent primers using the template cDNA and genomic DNA (gDNA), with RNase R treated or not. β-actin was used as an internal reference gene. **G** qRT-PCR analysis for circIPO7 reverse-transcribed by random primers or oligo dT primers. **H** Identification of circIPO7 cytoplasmic localization by FISH with or without RNase R treatment. Scale bars, 10 μM. **p < 0.01, ***p < 0.001, ****p < 0.0001.

To clarify the role of circRNAs in GC, we previously acquired the differential expression profile of circRNAs in patients with different prognoses by RNA sequencing and screened two circRNAs significantly related to postoperative recurrence of GC in the subsequent validation (*n* = 303) [18]. circIPO7, also known as hsa_circ_0005092, showed a higher expression level in GC patients with better outcomes. Herein, further correlation analysis of clinicopathological parameters suggested that the expression of circIPO7 was associated with the age, *N* stage and tumor differentiation of GC patients (Table 1). Patients with higher circIPO7 expression may be characterized by older age, fewer lymph node metastases and a higher degree of tumor differentiation. In addition, circIPO7 was downregulated in GC tissues and cells compared to paracarcinoma tissues and normal epithelial cells (Fig. 1A, B). These expression features raise the question of whether circIPO7 plays a role in GC development.

circIPO7 is generated from the back-splicing of exons 15-16 of the IPO7 gene with a full length of 290 nt, as shown in Fig. 1C. The back-spliced junction site of circIPO7 was amplified with divergent primers in cDNA but not genomic DNA (gDNA) and verified by Sanger sequencing (Fig. 1C, F). Multiple bioinformatics tools were used to analyze its sequence and structure. Figure 1D displays two predicted secondary structures of circIPO7 with a minimum free energy (MFE) of −57.9 kcal/mol. Further sequence alignment showed that human circIPO7 and mouse mmu-Ipo7_0007 (circAtlas ID) had 94.1% sequence similarity (Fig. 1E), illustrating its high sequence conservation. Exonuclease treatment was used to examine the stability of circIPO7, and the results suggested that circIPO7 was more resistant to digestion by RNase R than linear RNA (Fig. 1F, H). Meanwhile, circIPO7 was preferentially reverse transcribed by random 6 primers rather than oligo dT primers with a positive control of ciRS-7 (a typical circRNA) and negative control of its host gene, indicating that circIPO7 did not have a poly (A) tail (Fig. 1G and Supplementary Fig. S1A). Moreover, a FISH assay was performed with junction-specific probes and showed that circIPO7 was mainly located in the cytoplasm of GC cells (Fig. 1H). These results demonstrated the stability, conservation and cytoplasmic localization of circIPO7 as a circRNA.

### circIPO7 overexpression inhibited cell proliferation and tumor growth in GC

**Fig. 2:**
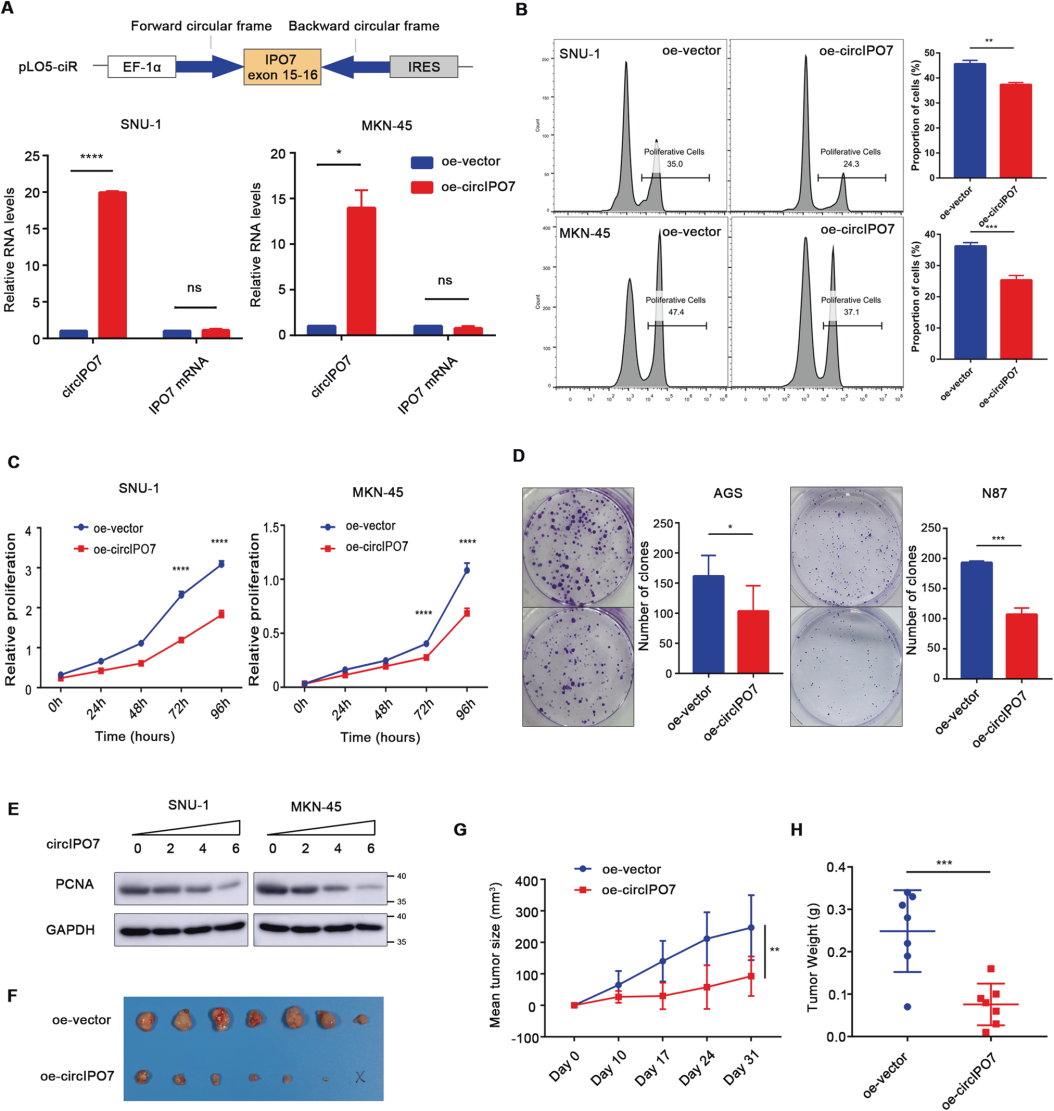
Role of circIPO7 in GC cell proliferation. **A** Schematic diagram of circIPO7 overexpression vector based on pLO5-ciR. qRT-PCR analysis was used to detect the overexpression efficiency of circIPO7 and IPO7 mRNA in SNU-1 and MKN-45 cells. oe, overexpression (in all figures). **B**–**D** Edu (**B**), CCK-8 (**C**), and clone formation (**D**) analysis of the effect of circIPO7 overexpression on the GC cell proliferation. **E** The changes of PCNA expression after the cells transiently transfected with gradient circIPO7 vector in SNU-1 and MKN-45 cells. **F**–**H** Xenograft models were used to analyze the effect of circIPO7 overexpression on tumor formation in vivo. The xenograft tumors were measured weekly, harvested after 4–5 weeks and weighed for analysis. ns no significant, *p < 0.05, **p < 0.01, ***p < 0.001, ****p < 0.0001.

Next, we further investigated the biological function of circIPO7 in GC development. Considering the low expression of circIPO7, we constructed several stable circIPO7-overexpressing GC cell lines using a lentivirus system, with no effect on its parental gene (Fig. 2A and Supplementary Fig. S1B). Compared with vector transfection, overexpression of circIPO7 in GC cells led to inhibition of cell viability, retardation of the DNA synthesis rate, and a more significant reduction in the size and numbers of colonies in vitro, suggesting the potential advantage of circIPO7 in suppressing cell proliferation in long-term effects (Fig. 2B–D and Supplementary Fig. S1C). Additionally, overexpression of circIPO7 induced a dose-dependent suppression of the proliferation-associated markers (PCNA) (Fig. 2E). To explore the effects of circIPO7 on the proliferation of GC cells in vivo, we constructed a subcutaneous nude mouse tumor model using MKN-45 cells. Overexpression of circIPO7 suppressed tumor growth in the subcutaneous tumor model compared with that in the control group, and lower tumor weights and volumes were detected in the overexpression group (Fig. 2F–H). Taken together, these results suggest that circIPO7 overexpression can inhibit GC cell proliferation in vitro and in vivo.

### circIPO7 directly bound with caprin-1

How does circIPO7 achieve the above biological functions? First, the parental gene-mediated regulatory mechanism was excluded in our study, as circIPO7 overexpression had no effect on the protein and mRNA levels of IPO7 (Fig. 2A and Supplementary Fig. S2A). Subsequently, bioinformatics prediction and preliminary verification were used to evaluate the function of circIPO7 as a miRNA sponge, protein scaffold, modulator of transcription and translation template. We confirmed that the sequence of circIPO7 had the potential to bind proteins but lacked sufficient miRNA binding sites and translational elements (Supplementary Fig. S2B–E).

**Fig. 3:**
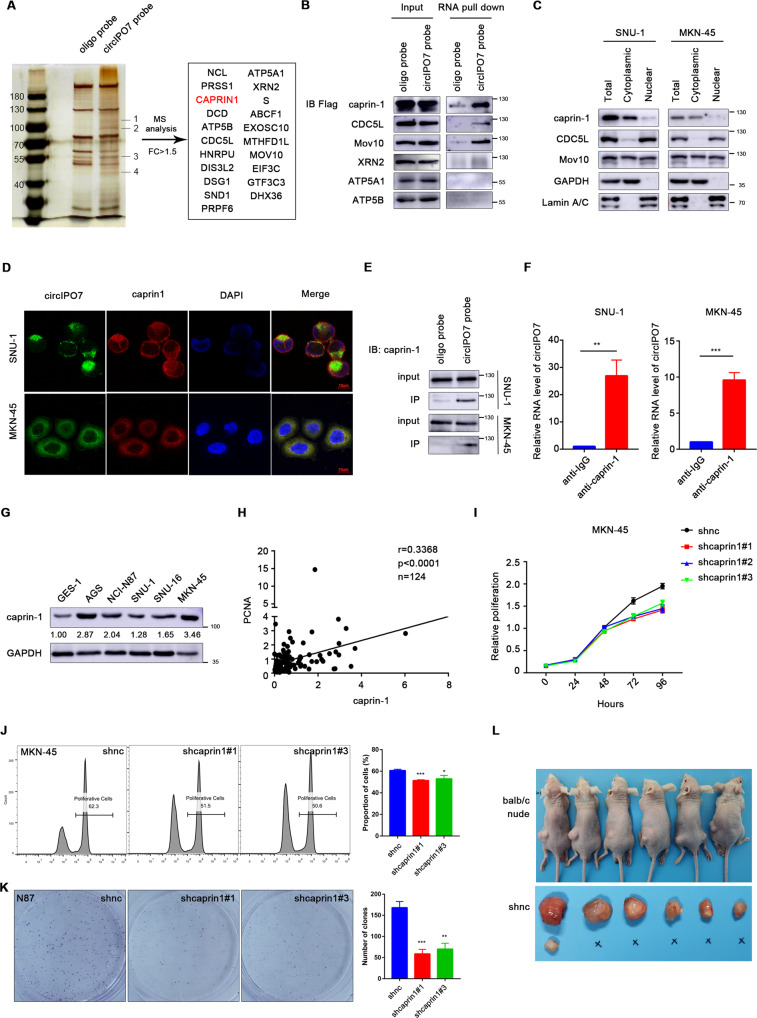
Identification of the interaction between circIPO7 and caprin-1. **A** Identification of proteins that interact with circIPO7 by RNA pull-down, silver staining and mass spectrometry (MS). FC fold change. **B** For the screened proteins, the overexpressing vectors with flag tag was constructed and transfected into 293 T cells. Then, RNA pull-down assay was used to verify the interaction between circIPO7 and these exogenous proteins. **C** The subcellular components of GC cells were isolated *via* a NE-PER™ nuclear and cytoplasmic extraction kit, and then western blot was used to measure the localization caprin-1, CDC5L and Mov10. GAPDH, cytoplasm control. Lamin A/C, nuclear control. **D** Intracellular localization of circIPO7 and caprin-1 was performed by FISH analysis. Scale bars, 10 μM. **E** RNA pull-down assay verified the interaction between circIPO7 and endogenous caprin-1 in GC cells. **F** RIP assay verified the interaction between circIPO7 and endogenous caprin-1 in GC cells. **G** Caprin-1 expression in normal epithelial cell (GES-1) and tumor cell lines (SNU-16, AGS, NCI-N87, SNU-1 and MKN-45) were examined by western blot. The density of bands was quantified with ImageJ. **H** Pearson correlation analysis between the expression of caprin-1 and PCNA in GC tissue (*n* = 124, *p* < 0.0001). **I**–**K** CCK-8 (**I**), Edu (**J**), and clone formation (**K**) analysis of the effect of caprin-1 knockdown on the GC cell proliferation. **L** Xenograft models were used to analyze the effect of caprin-1 knockdown on tumor formation in vivo. **p < 0.01, ***p < 0.001, ****p < 0.0001.

Through RNA pull-down assays and mass spectrometry, a total of 21 proteins that may specifically interact with circIPO7 were identified with the criteria of circIPO7 probe/oligo probe >1.5 (Fig. 3A and Supplementary Table S2). These proteins were mainly RBPs that are involved in RNA splicing, transport, translation and degradation, while others play roles in mitochondrial energy metabolism and cell division. Considering the function of circIPO7 in cell proliferation, we selected 6 candidate proteins, constructed their overexpression vectors, and then transfected them into 293 T cells. An RNA pull-down assay was performed to verify their interaction with circIPO7 and showed that caprin-1 strongly bound to circIPO7 (Fig. 3B). Furthermore, western blot and RNA FISH analysis revealed that caprin-1 was colocalized in the cytoplasm of GC cells with circIPO7, which is more conducive to their interaction (Fig. 3C, D). Subsequently, we found that endogenous caprin-1 could be pulled down by a probe specifically targeting circIPO7 (Fig. 3E), and in turn, circIPO7 could be precipitated by the anti-caprin-1 antibody in MKN-45 and SNU-1 cells (Fig. 3F), which may be enhanced with the overexpression of circIPO7 (Supplementary Fig. S3A). These results supported the interaction between circIPO7 and caprin-1.

### Expression characteristics and biological function of caprin-1 in GC

Caprin-1 has been reported to be a regulator of mRNA translation and is abnormally expressed in various cancers [15, 19]. In our study, caprin-1 was highly expressed in GC cells and tissues (Fig. 3G and Supplementary Fig. S3B, C). Pearson correlation analysis identified a positive correlation between the expression level of caprin-1 and PCNA at the tissue level (*n* = 124, *p* < 0.0001, Fig. 3H), suggesting the involvement of caprin-1 in cell proliferation. To verify this finding, we knocked down caprin-1 in GC cells *via* shRNAs. Functional experiments showed that silencing caprin-1 significantly inhibited PCNA expression, cell viability, DNA synthesis rate, and clone formation ability of GC cells in vitro (Fig. 3I–K and Supplementary Fig. S3D–G), which is consistent with the results of circIPO7 overexpression. Moreover, subcutaneous injection of the caprin-1-silenced MKN-45 cells in nude mice significantly suppressed tumor formation and the growth rate compared to those in the control group (Fig. 3L). Collectively, caprin-1 and circIPO7 have opposite effects on cell proliferation, and circIPO7 may serve as a tumor suppressor by binding caprin-1.

### circIPO7 bound caprin-1 and competed with G3BP1

**Fig. 4:**
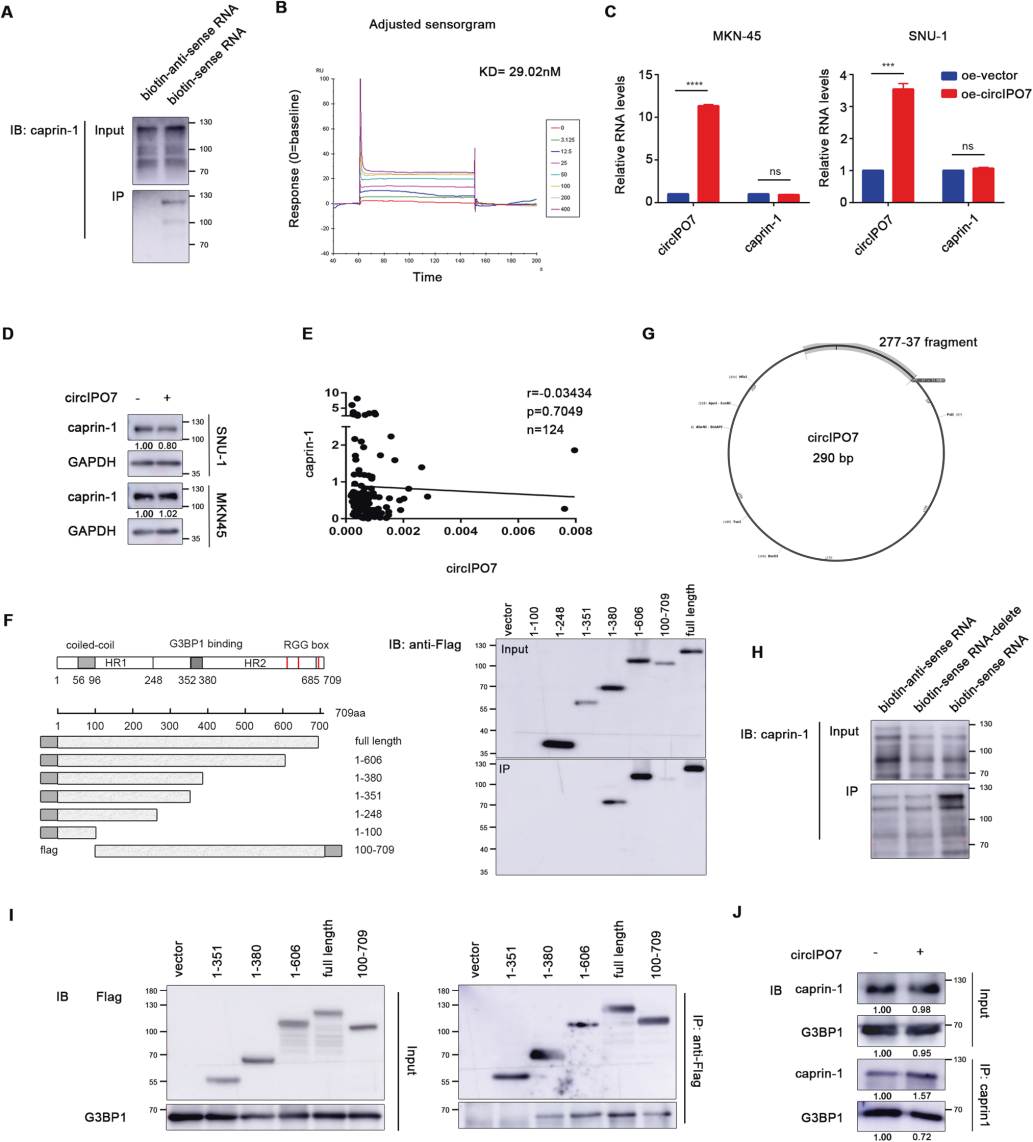
Analysis of the binding sits of circIPO7 on caprin-1. **A** Biotinylated sense and antisense linearized circIPO7 that transcribed in vitro were mixed with purified caprin-1 recombinant protein, and their direct interaction was analyzed by RNA pull-down assay. **B** The direct interaction between circIPO7 and caprin-1 and related kinetic parameters were analyzed by BIAcore surface plasmon resonance (SPR) biosensor. Biotinylated linearized circIPO7 was immobilized to streptavidin sensor surface, and incubated with increasing concentrations (0–400 nM) of purified caprin-1. The association and disassociation of caprin-1 was monitored by BIAcore as an interference wavelength shift. **C**, **D** The expression of caprin-1 mRNA (**C**) and protein (**D**) were examined in GC cells transfected with vector or circIPO7. **E** Pearson correlation analysis between the expression levels of circIPO7 and caprin-1 in 124 GC tissues. **F** Schematic diagram of truncated caprin-1 vectors. The truncated caprin-1 vectors were transfected into GC cells, and the binding site of circIPO7 on caprin-1 was analyzed by RNA pull-down assay. **G** Prediction of the binding site of circIPO7 to caprin-1 via catRAPID. **H** Biotinylated antisense circIPO7, sense circIPO7 or sense circIPO7-delete (circIPO7 without 277-37 fragment) that transcribed in vitro were mixed with purified caprin-1 recombinant protein, and their direct interaction was analyzed by RNA pull-down assay. **I** The truncated caprin-1 vectors were transfected into GC cells, and the binding site of G3BP1 on caprin-1 was analyzed by co-IP assay. **J** RIP assay was used to analyze the effect of circIPO7 overexpression on the interaction between caprin-1 and G3BP1. ns no significant, ***p < 0.001, ****p < 0.0001.

Moreover, to clarify whether the interaction between circIPO7 and caprin-1 is direct or not, we synthesized biotinylated linearized circIPO7 with a retained junction site and purified full length caprin-1 recombinant protein, followed by an RNA pull-down assay. As a result, these two engineered molecules bound to each other during the coincubation in vitro (Fig. 4A). A BIAcore surface plasmon resonance (SPR) biosensor was further used to monitor their association and disassociation, with a KD of 29.02 nM (Fig. 4B), further supporting the direct interaction between circIPO7 and caprin-1.

Next, we further analyzed the regulatory mechanism of circIPO7 on caprin-1. First, circIPO7 overexpression was proven to have no effect on caprin-1 mRNA and protein levels (Fig. 4C–E), suggesting that circIPO7 likely regulates the downstream pathway of caprin-1. To clarify the key domain of caprin-1 contributing to the interaction with circIPO7, a series of truncations were transfected into SNU-1 cells. RNA pull-down assays revealed that the loss of amino acids 1–100 and 352–380 on caprin-1 decreased its binding affinity with circIPO7, and the latter was decisive (Fig. 4F). Considering the importance of the α-coiled coil present in amino acids 1–100 in providing a docking surface for nucleic acids, we speculated that amino acids 1–100 on caprin-1 was favorable but not necessary for its binding to circIPO7. In addition, we used catRAPID to predict the binding site of circIPO7 and caprin-1, and found that the RNA binding domain PF12287 of caprin-1 (amino acids 356–386, which is coincident with the result of RNA pull down assay) and the 277-37 fragment on the back-splicing junction sequence (BSJ) of circIPO7 promoted their combination, as shown in Fig. 4G and Supplementary Table S3. RNA pull-down assay in vitro was further performed by caprin-1 recombinant protein and biotinylated linearized circIPO7 or circIPO7 without 277-37 fragment, showing that the loss of 277-37 fragment on circIPO7 declined its combination to caprin-1 (Fig. 4H). All the above results supported that amino acids 352–380 on caprin-1 and 277-37 fragment on circIPO7 were their direct binding sites.

Interestingly, previous studies reported that the amino acids 352–380 of caprin-1 also mediated its interaction with G3BP1, the most common molecular chaperone for caprin-1, which was confirmed in our study by co-IP assays (Fig. 4I). That is, the binding sites of circIPO7 and caprin-1 overlapped with those of caprin-1 and G3BP1. Expectedly, we found a decreased interaction between caprin-1 and G3BP1 when circIPO7 was over-expressed (Fig. 4J). These results implied that circIPO7 might block the binding of caprin-1 and G3BP1 through competitive inhibition.

### circIPO7 inhibited G3BP1-mediated recruitment of caprin-1 to ribosomes

**Fig. 5:**
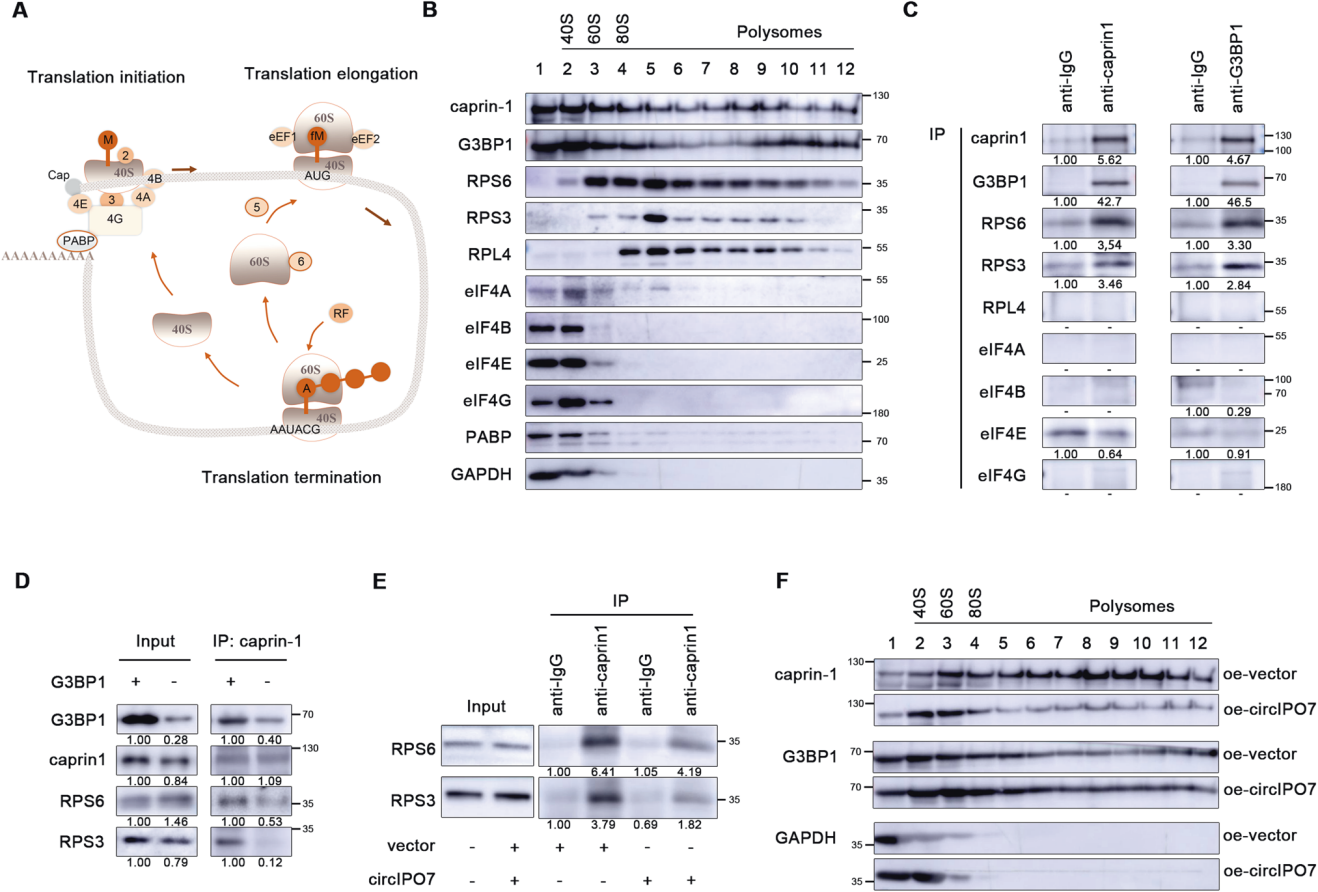
Effect of circIPO7 on caprin-1 downstream translational regulation. **A** Schematic diagram of mRNA translation. (1) Translation initiation. The ribosomal 40 S subunit binds to the ternary complex (eIF2-GTP-tRNA ^Met^) and other factors, including eIF-3, to form pre-initiation complex, and locates to the 5’ cap structure of mRNA under the influence of capbinding complex including eIF4A, eIF4E, and eIF4G. After assembling with mRNA, the translation initiation complex scans the mRNA from the 5’ end to the first start codon (AUG), and then recruit 60 S ribosomes to form active 80 S ribosomes. (2) Translation elongation. The formation of 80 S ribosome produces two sites for binding to tRNAs: P-site and A-site. Aminoacyl-tRNA enters A-site, followed by the release of amino acid of tRNA^Met^ located on P-site and peptide bond formation. Subsequently, elongation factor, eEF1 and eEF2, are involved in tRNA introduction and ribosomal translocation respectively. (3) Translation termination. When 80 S ribosomes meets stop codons (UAG, UGA, UAA), release factor (RF) rather than aminoacyl-tRNA binds to the A-site, leading to the termination of mRNA translation. Eukaryotic translation initiation factors (eIF2, eIF3, eIF4A, eIF4B, eIF4E, eIF4G, eIF5) were denoted by abbreviations. EF, elongation factor. RF, release factor. **B** Total of 12 ribosomal components were isolated by sucrose gradient centrifugation. The distribution of caprin-1, G3BP1 and other ribosomal proteins were analyzed by western blot. **C** The interaction between caprin-1 and G3BP1 with ribosomal proteins was analyzed by co-IP. **D** The effect of G3BP1 knockdown on the interaction between caprin-1 and ribosomal proteins (RPS6 and RPS3) was analyzed by co-IP assay. **E** The effect of circIPO7 overexpression on the interaction between caprin-1 and ribosomal proteins (RPS6 and RPS3) was analyzed by co-IP assay. **F** Ribosomal profiling analysis of caprin-1 and G3BP1 distribution upon circIPO7 overexpression.

Studies have demonstrated that the complex formed by caprin-1 and G3BP1 is involved in the translational regulation of cell division-related mRNAs [14, 15]. Ribosomes are known to play an active and passive role in protein synthesis (Fig. 5A). To elucidate whether circIPO7 regulates the downstream translation-related mechanisms of caprin-1, we conducted polysome profiling analysis followed by western blotting. Ribosomes were size-fractionated by sucrose density gradient centrifugation and divided into small (40 S) and large (60 S) ribosomal subunits, monosomes (80 S), and polysomes. As shown in Fig. 5B, with a control of GAPDH, caprin-1 and G3BP1 were detected in all ribosomal components, sharing a similar distribution with the 40 S ribosomal subunit proteins RPS6 and RPS3. Moreover, we discovered their interaction by a co-IP assay (Fig. 5C). Considering the combination and colocalization between G3BP1 and the ribosomal 40 S subunit as reported previously [20], we hypothesized that the interaction between caprin-1 and 40 S ribosomal proteins was indirect and bridged by G3BP1. In fact, G3BP1 knockdown significantly decreased the binding of caprin-1 with RPS3 and RPS6 (Fig. 5D). Consistently, circIPO7 overexpression had the same effect as G3BP1 knockdown on the interaction of caprin-1 with RPS3 and RPS6, resulting in the deficiency of caprin-1 in polyribosomal components rather than G3BP1 (Fig. 5E, F). These results implied that circIPO7 could competitively bind with caprin-1 and then inhibit its enrichment in ribosomes, which may further result in the translational inhibition of its target mRNAs.

### Identification of caprin-1 target mRNAs related to cell proliferation

**Fig. 6:**
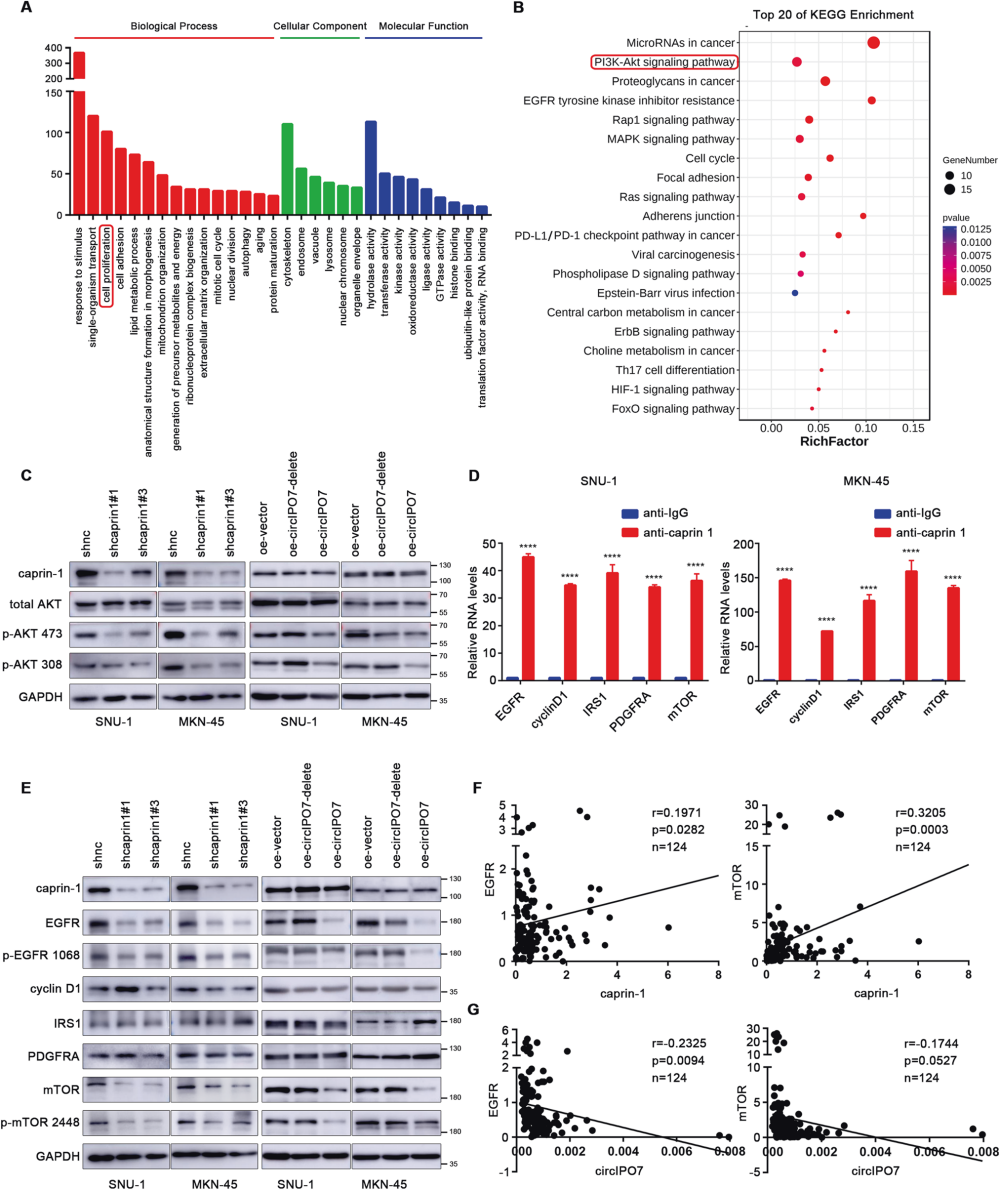
Identification of downstream cell proliferation-related mRNAs of caprin-1. **A** The mRNAs that may bind to caprin-1 was identified by RIP sequencing, followed by GO enrichment analysis. **B** KEGG pathway enrichment was performed to analyze the top 20 pathways of these cell proliferation-related mRNAs selected from GO enrichment analysis. **C** The effects of caprin-1 knockdown and circIPO7 overexpression on the phosphorylation of Akt were analyzed by western blot. The group of overexpressing circIPO7 without 277-37 fragment (defined as circIPO7-delete in all figures) was also used as negative control. **D** RIP assay was used to verify the interaction between caprin-1 and selected mRNAs (EGFR, cyclin D1, IRS1, PDGFRA and mTOR) obtained from RIP sequencing. **E** The effects of caprin-1 knockdown and circIPO7 or circIPO7-delete overexpression on the protein level of above 5 molecules were analyzed by western blot. **F** Pearson correlation analysis between the expression levels between EGFR, mTOR with caprin-1 (*n* = 124). **G** Pearson correlation analysis between the expression levels between EGFR, mTOR with circIPO7 (*n* = 124). ****p < 0.0001.

To verify our hypothesis, we performed an RIP sequencing assay to investigate the potential target mRNAs of caprin-1. After genome alignment and IP/input standardization, a total of 770 mRNAs that may interact with caprin-1 were identified and subsequently analyzed for GO and KEGG enrichment (Fig. 6A, B and Supplementary Table S4). These mRNAs were mainly involved in response to stimulus, cell proliferation, cell adhesion, and lipid metabolic processes. We focused on 105 mRNAs related to cell proliferation and found that the PI3K-Akt signaling pathway is one of the top enriched pathways. Akt phosphorylation was inhibited in GC cells with caprin-1 knockdown or circIPO7 overexpression, indicating that circIPO7 and caprin-1 could regulate the activation of the PI3K-Akt pathway in the opposite direction (Fig. 6C). Eventually, the top 5 molecules (EGFR, PDGFRA, IRS1, CCND1 and MTOR) with more binding sites for caprin-1, which were enriched in the PI3K-Akt signaling pathway and identified by the RIP assay, were selected as candidates for further validation (Fig. 6D and Supplementary Table S5).

### circIPO7 inhibited EGFR and mTOR expression and cell proliferation by binding to caprin-1

Next, we knocked down caprin-1 in MKN-45 and SNU-1 cells and observed a significant decrease in the protein expression and phosphorylation levels of EGFR and mTOR without a corresponding reduction at the mRNA level, similar to the effects of circIPO7 but not circIPO7-delete overexpression (Fig. 6E and Supplementary Fig. S4). In addition, Pearson correlation analysis showed that the expression levels of EGFR and mTOR in GC tissues were positively correlated with caprin-1 (EGFR, *p* = 0.0282; mTOR, *p* = 0.0003) but negatively correlated with circIPO7 (EGFR, *p* = 0.0094; mTOR, *p* = 0.0527) (Fig. 6F–G and Supplementary Fig. S5), further demonstrating the opposite effect of circIPO7 and caprin-1 on their expression.

**Fig. 7:**
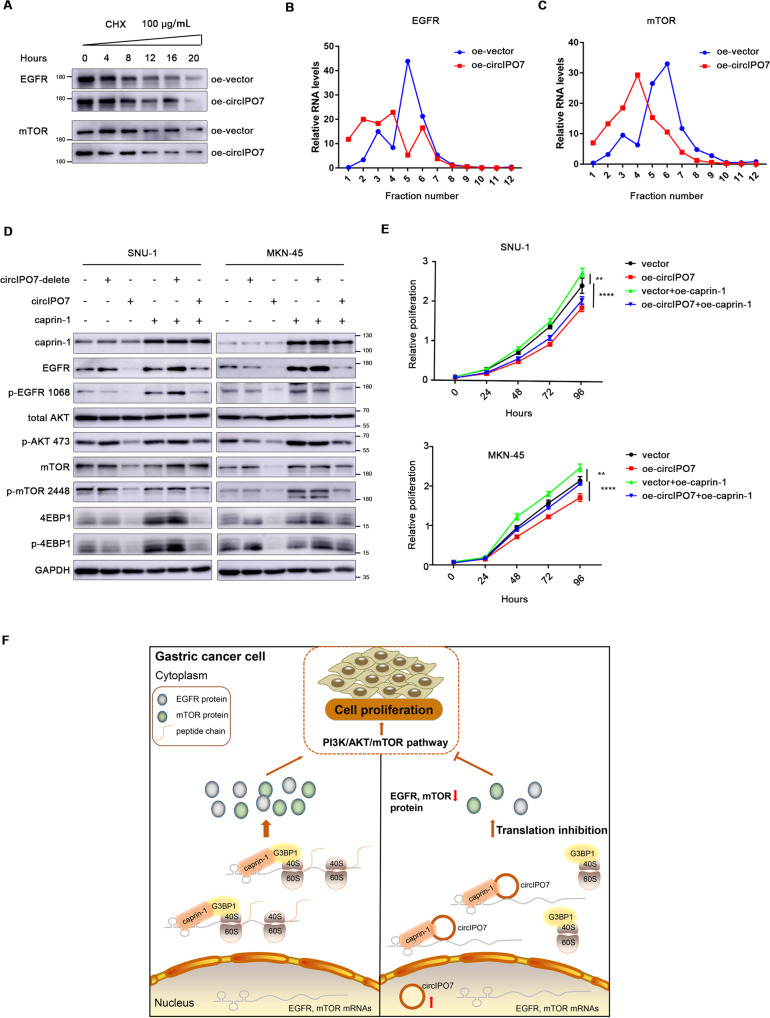
Effect of the circIPO7-caprin-1 complex on the mRNA translation of EGFR and mTOR. **A** GC cells with vector- or circIPO7-overexpression were treated with CHX for different times (0 h, 4 h, 8 h, 12 h, 16 h), and then lysed to detect the protein levels of EGFR and mTOR by western blot. **B**, **C** Ribosomal profiling analysis of EGFR mRNA (b) and mTOR mRNA (c) distribution in GC cells with vector- or circIPO7-overexpression. **D** The effects of circIPO7 or/and caprin-1 overexpression on the PI3K/AKT/mTOR pathway were analyzed by western blot. **E** CCK-8 analysis of the effect of circIPO7 or/and caprin-1 overexpression on GC cell proliferation. **F** Schematic model depicting the mechanism of circIPO7 in suppressing GC cell proliferation. In GC cells, caprin-1 interacts with G3BP1 and enters the translation initiation complex, facilitating the translation of its target mRNAs (EGFR, mTOR). Overexpressed circIPO7 could competently bind caprin-1 with G3BP1, and then block the recruitment of caprin-1 along with its target mRNAs to 40 S ribosomal subunit, leading to the inhibition of their translation and PI3K/AKT/mTOR signaling activation, thus suppressing GC cell proliferation ultimately.

To address whether the observed changes in protein levels were elicited at the mRNA translation level, we added CHX to cell cultures to block protein synthesis and found that the degradation rates of the EGFR and mTOR proteins were equivalent in both the vector- and circIPO7-overexpressing cells (Fig. 7A and Supplementary Fig. S6A). In addition, MG132 was used to inhibit protein degradation, resulting in a more significant decrease in EGFR and mTOR protein levels in circIPO7-overexpressing cells (Supplementary Fig. S6B). Furthermore, polysome profiling analysis showed that the distribution of EGFR and mTOR mRNAs transferred from the heavier to the lighter polyribosomal components upon circIPO7 overexpression, while global RNA did not show this pattern (Fig. 7B, C and Supplementary Fig. S6C). These results suggested that the changes in the EGFR and mTOR proteins, as initially suspected, were mainly caused by post-transcriptional regulation of mRNA translation.

Finally, we conducted a rescue assay to investigate whether the effect of circIPO7 on EGFR and mTOR protein levels was associated with caprin-1. The results showed that caprin-1 overexpression inhibited circIPO7-induced decreases in the EGFR and mTOR protein levels and the activation of the PI3K/AKT/mTOR pathway, followed by an escalation of GC cell proliferation (Fig. 7D, E). Taken together, our data suggested that circIPO7 blocked the recruitment of caprin-1 and its target mRNAs to ribosomes, which resulted in the inhibition of GC cell proliferation *via* the PI3K/AKT/mTOR pathway (Fig. 7F).

## Discussion

CircRNAs were first identified in RNA viruses and defined as “splicing noise” because of their low expression [21]. Recently, with the development of experimental technologies, their ubiquity and function have been gradually revealed. In GC, for example, circNRIP1 was proven to sponge miR-149-5p to affect AKT1 expression and was transmitted in cells through exosomes, followed by enhanced tumor metastasis in PDX models [22]. Circ-HuR, derived from human antigen R, was downregulated in GC cells and tissues. Overexpressed circ-HuR interacted with CNBP and inhibited its binding to the HuR promoter, further leading to a decline in HuR expression and suppression of tumor progression [23]. circIPO7 was previously shown to have a positive relationship with the prognoses of GC patients, and herein, we further discovered that overexpressed circIPO7 in GC cells could directly bind with caprin-1 and then block its recruitment to ribosomes by competing against G3BP1, thereby leading to translational inhibition of its target mRNAs, including EGFR and mTOR. As a consequence, this molecule substantially suppressed the activation of the PI3K/AKT/mTOR pathway and cell proliferation in vitro and in vivo.

As reported, protein scaffolds, especially for RBPs, are one of the most common functions of circRNAs. RBPs are involved in all aspects of RNA metabolism, including RNA splicing, transport, stabilization, localization and translation [24]. The tertiary structure of circRNAs is more favorable for binding to RBPs than linear RNAs [25], facilitating the potential of circRNAs as protein interaction platforms. Generally, circRNAs localized in the cytoplasm preferentially serve as miRNA sponges to regulate gene expression and bind to RBPs to affect their location and function, while those located in the nucleus regulate transcription [26]. circIPO7, an exonic circular RNA (ecircRNA) mainly located in the cytoplasm, was proven to directly bind with carpin-1 and thus regulate RNA metabolism at the post-transcriptional level in our study.

Caprin-1 has been reported to be abnormally expressed in various cancers. Mechanistically, the complex containing caprin-1 and G3BP1 is involved in the translational regulation of cell division-related mRNAs, such as c-Myc and Ccnd2 [14, 15]. In addition, caprin-1 was reported to interact with fragile X mental retardation protein (FMRP) and colocalize in the polysomes [27], indicating its role in mRNA translation. circIPO7 had no effect on caprin-1 expression, prompting us to uncover its possible regulatory pathway. Many studies have shown that RBPs can reshape mRNA structure and alter mRNA location to regulate their affinity for translation machinery [28]. Herein, knockdown of caprin-1 in GC cells resulted in a significant blockage of malignant cell proliferation and tumor incidence, in line with circIPO7 overexpression. Intriguingly, circIPO7 was shown to compete with G3BP1 for binding to caprin-1 with a shared sequence of caprin-1, which provides novel insights into the potential role of the circIPO7-caprin-1 complex in mRNA translation.

mRNA translation is a complex process and can be divided into three main steps, translational initiation, elongation and termination, which are induced by the orderly combination and dissociation of ribosomes and translation-related proteins on mRNAs [29]. Polysomes formed by multiple tandem ribosomes on mRNA increase the synthesis efficiency of peptide chains. Dysregulation of translation may lead to aberrant proliferation, differentiation, apoptosis, angiogenesis and malignant transformation. Previous studies have demonstrated that in addition to translation initiation factors, the efficiency of mRNA translation could be affected by RBPs and noncoding RNAs [30–32]. Among them, circRNAs not only independently regulate translation but also interact with RBPs to regulate initiation of translation. In this study, we observed a similar distribution between caprin-1 and ribosomal 40 S subunit proteins (RPS3 and RPS6) in the polysome fractions, as well as their notable interaction, which could be blocked by G3BP1 knockdown and circIPO7 overexpression. That is, the recruitment of caprin-1 to ribosomes was induced by G3BP1, and in contrast, overexpressed circIPO7 isolated caprin-1 from ribosome-rich areas by competing with G3BP1. Given the function of caprin-1 as an RBP, it is reasonable to presume that their combination and dissociation with the translation initiation complex will inevitably lead to the translational activation and inhibition of its target mRNAs.

To verify our hypothesis, we performed RIP sequencing to explore the RNA-binding profile of caprin-1 and screened 2 target mRNAs, EGFR and mTOR, which are enriched in the PI3K-AKT pathway, a key pathway involved in cell proliferation. Their expression is oppositely regulated by caprin-1 and circIPO7, which occurs during mRNA translation rather than mRNA transcription or protein degradation, since caprin-1 along with its target mRNAs are transferred from the heavier to the lighter polysome fractions upon circIPO7 overexpression. Notably, circIPO7 overexpression did not affect the distribution profile of total RNAs, as we reported, indicating that the mRNAs regulated by the circIPO7-caprin-1 complex are selective. The distinctive RNA motifs that caprin-1 preferentially binds were not investigated in the current study. GO functional enrichment of caprin-1 target mRNAs revealed that, in addition to cell proliferation, caprin-1 may regulate other physiological processes, including response to stimulus, cell adhesion, lipid metabolic process, etc. Cell proliferation may be only one aspect or a comprehensive manifestation of the circIPO7-caprin-1 complex on cellular function, and further investigation is still needed. Nevertheless, our study on the regulatory mechanism of circIPO7 and caprin-1 facilitates a better understanding of GC carcinogenesis.

In conclusion, our study uncovered a novel mechanism by which circIPO7 directly bound caprin-1 to prevent G3BP1-induced recruitment to ribosomes and then silenced the mRNA translation of EGFR and mTOR, as well as the PI3K/AKT/mTOR signaling pathway, ultimately leading to inhibition of cell proliferation. Our findings provide a molecular basis for new insights into the important roles of circRNAs in GC and offer some hints for the development of therapeutic targets for cancer intervention.

## Methods

### Patients and samples

All samples (59 paired tumor and paracancinoma tissues and 124 separate tumor tissues) from GC patients were collected from Tianjin Medical University Cancer Institute and Hospital. The detailed clinicopathological characteristics of the patients are shown in Table 1. Our study was approved by the Ethics Committee of Tianjin Medical University Cancer Institute and Hospital.

**Table 1:** Correlation between circIPO7 expression level and clinicopathological parameters in GC patients.

Variables	Group	Cases *n* = 303	circIPO7 expression		*P* value
			High	Low	
			*n* = 151	*n* = 152	
Age	≤60 years	155	71	90	0.0335*
	>60 years	148	80	62	
Sex	Male	222	111	111	0.9242
	Female	81	40	41	
Tumor size	≤5 cm	143	75	68	0.3815
	>5 cm	150	71	79	
	unmentioned	10	5	5	
T stage	T1-3	41	23	18	0.6311
	T4a	181	90	91	
	T4b	82	38	43	
N stage	N0	73	33	40	0.0428*
	N1	73	44	29	
	N2	76	42	34	
	N3	81	32	49	
TNM stage	I-II	75	36	39	0.103
	IIIA	113	66	47	
	IIIB	63	26	37	
	IIIC	52	22	29	
Differentiation	Poor-undifferentation	158	69	89	0.0251*
	Well-moderate differentation	145	82	63	

*p < 0.05.

### Cell culture

The human gastric mucosal epithelial cell line (GES-1), GC cell lines (AGS, NCI-N87, MKN-45, SNU-1, and SNU-16), and HEK293T cells were purchased from ATCC and authenticated by STR profiling. These cells were cultured in F12K, RPMI 1640 or DMEM (Gibco, USA) medium with 10% FBS (Gibco) and 1% penicillin-streptomycin at 37 °C in a 5% CO_2_ atmosphere.

### Lentivirus-mediated stable cell line construction

For generation of the circIPO7 overexpression system, full-length human circIPO7 cDNA was synthesized and cloned into the pLC5-ciR vector (Geneseed, China) with a negative control empty vector and then verified by Sanger sequencing. Small hairpin RNAs (shRNAs) were used to specifically silence caprin-1. Stable circIPO7 overexpression or caprin-1 knockdown in GC cells was achieved *via* transduction with lentivirus, which was generated by transfecting HEK293T cells with packaging plasmid according to the manufacturer’s protocol, in the presence of 8 μg/mL polybrene (Sigma, USA). Subsequently, the cells were selected with 2 μg/ml puromycin (Invitrogen, USA) for 2 weeks.

### Quantitative reverse transcription-polymerase chain reaction (qRT-PCR) assay

Total RNA was extracted from cells with TRIzol reagent (Invitrogen, USA) according to the manufacturer’s instructions. A total of 500 ng of total RNA was subjected to reverse transcription and quantitative PCR using the PrimeScritTM RT reagent kit (TaKaRa, China) and TB Green Premix Ex Taq II (TaKaRa, China). Both relative and absolute RNA quantitation was performed in our study, and β-actin was used as an internal reference gene. The primer sequences are listed in Supplementary Table S1.

### Fluorescence in situ hybridization (FISH)

Biotin-labeled RNA probes targeting circIPO7 back-splice junctions were designed and synthesized by GenePharma (Shanghai, China). GC cells were grown to 50–75% confluence at the time of fixation with 4% paraformaldehyde and hybridized with circIPO7 probes at 37 °C overnight in hybridization buffer (10% formamide, 10% dextran sulfate, 2×SSC), followed by incubation with Alexa Fluor 488-conjugated streptavidin (Invitrogen, USA) in the dark for 1 h. Finally, the cells were counterstained with DAPI (Invitrogen, USA) for nuclear visualization. Signals were detected under a Zeiss LSM880 confocal microscope (Leica Microsystems, Germany). The sequences of the probes were as follows: circIPO7, 5’-GGUGUGAUAUAUUCUUUAGCUCUUUAUGAUCUUCAAC-3’; random control 5’-AGCUGAAUCAUGAGAACCAGAUC-3’.

### RNase R treatment

A total of 2 μg RNA from GC cells was treated with or without 3 U/μg RNase R (Epicenter Technologies, USA) for 15 min at 37 °C in a volume of 20 μl. Then, the RNA was extracted with phenol/chloroform followed by ethanol precipitation. An equal volume of treated RNA was used for reverse transcription, PCR amplification and agarose gel electrophoresis. β-actin was used as an internal reference gene.

### Western blot

Protein levels were examined by western blot analysis. In brief, proteins lysed from cells were separated by SDS-electrophoresis and then transferred to a PVDF membrane. The membrane was blocked and incubated with primary antibodies. Antibodies against caprin-1 and G3BP1 were purchased from Proteintech (Wuhan, China). Antibodies against PCNA, pan-AKT, phospho-AKT Thr308, phospho-AKT Ser473, cyclin D1, mTOR, phospho-mTOR Ser2448, 4EBP1, and phospho-4EBP1 were purchased from Cell Signaling Technology (Danvers, USA). Antibodies against EGFR, phospho-EGFR Tyr1068, RPS3, RPS6, RPL4, eIF4A, eIF4B, eIF4E, eIF4G, PABP, IRS1, and PDGFRA were purchased from Abcam (Cambridge, USA). HRP-conjugated anti-rabbit and anti-mouse IgG antibodies (Cell Signaling Technology, USA) were used as the secondary antibodies. Protein detection was performed with a chemiluminescence system (Bio-Rad, USA).

### Cell proliferation assay

We utilized a Cell Counting Kit-8 (CCK-8), 5-ethynyl-2’-deoxyuridine (EdU), and a colony formation assay to monitor GC cell proliferation according to the manufacturer’s protocols. For the CCK-8 assay, 3 × 10^3^ modified cells were cultured in each well of 96-well plates for the indicated times (0 h, 24 h, 48 h, 72 h, and 96 h), and ATP activity was detected to measure cell viability. For the EdU assay, 1 × 10^4^ modified cells were cultured in 96-well plates overnight, and then, Edu solution (10 μM) was added for 2 h. After fixation, permeabilization and nucleic acid staining, the DNA synthesis of cells was evaluated by flow cytometry (BD Biosciences, FACSCanto II, USA). For the colony formation assay, 800 modified cells were cultured in 6-well plates for 2 weeks. After fixation and staining with crystal violet, the number of clones was counted to determine the cloning ability of the cells.

### Xenograft models

Five-week-old male BALB/c-nude mice were purchased from Charles River (Beijing, China) and maintained under pathogen-free conditions. These mice (*n* = 6 or 7) were injected subcutaneously into both sides with 5 × 10^5^ modified MKN-45 cells in 100 µL of PBS. The tumor volume was measured weekly and calculated with the following formula: volume (mm^3^) = length × width^2^/2. The xenograft tumors were harvested after 4–5 weeks and weighed for analysis. All experiments were conducted in accordance with the guidelines and approved by the Animal Ethical and Welfare Committee of Tianjin Medical University Cancer Institute and Hospital.

### RNA pull-down assay

The biotin-labeled RNA probe and control probe were in accordance with those used in the FISH assay. Modified cells (1 × 10^7^) were transfected with biotin-labeled probes for 24 h and then lysed in IP buffer containing RNase inhibitor. The lysates were incubated with 50 µL of Streptavidin C1 magnetic beads (Invitrogen, USA) at room temperature for 30 min and then washed briefly with IP buffer, followed by western blotting and qRT-PCR.

### RNA immunoprecipitation (RIP)

The RIP assay was performed using an EZ Magna RIP Kit (Millipore, USA) according to the manufacturer’s protocol. In brief, 2 × 10^7^ modified GC cells were lysed and incubated with antibodies, as well as magnetic beads at 4 °C overnight, with an IgG antibody control. The immunoprecipitated RNA was extracted and purified and then analyzed by qRT-PCR.

### Synthesis of recombinant caprin-1 protein and linear circIPO7

For synthesis of the caprin-1 recombinant protein, caprin-1 cDNA was amplified by PCR and cloned into the pET28a (+)-His6 vector. This vector was transformed and expressed in *Escherichia coli* BL21(DE3). Bacteria were induced with 0.5 mM isopropyl-β-d-thiogalactopyranoside (IPTG) for 12 h at 30 °C with shaking, and then lysed in bacterial lysis buffer (25 mM Tris-HCl, pH 7.5, 150 mM NaCl, 1% Triton X-100, and 1 mM PMSF) by sonication on ice. The supernatant was collected by centrifugation at 10,000 × *g* for 30 min at 4 °C and then flowed through HisTALON Gravity Columns (Clontech, USA). The columns were washed and eluted according to the manufacturer’s protocol. SDS-PAGE followed by Coomassie Brilliant Blue staining was used to detect the molecular weight and purity of caprin-1 recombinant protein. circIPO7 was linearized at the opposite site of the back-spliced junction point and cloned into the pUC57-Amp vector. Synthesis, biotin labeling and purification of linear circIPO7 was performed using the Pierce™ RNA 3’ End Desthiobiotinylation Kit (Invitrogen, USA) according to the manufacturer’s protocol.

### Polysome profiling analysis

A sucrose density gradient assay was used to analyze mRNA distribution and translation profiles as described previously [33]. In brief, modified cells exposed to cycloheximide (CHX, 100 μg/mL) for 15 min were lysed in polysome extraction buffer containing 20 mM Tris-HCl (pH 7.5), 100 mM KCl, 5 mM MgCl_2_, 0.5% CA-630, 1 × protease inhibitor, RNase inhibitor and 100 μg/mL CHX. The polysome lysates were centrifuged at 12,000 × *g* for 10 min at 4 °C to pellet the nuclei and debris. Equal amounts of supernatant were loaded on top of a prepared 10 to 50% sucrose gradient and then extracted into twelve polysome fractions from top to bottom by ultracentrifugation at 39,000 rpm for 1.5 h at 4 °C. The RNA amount of each fraction was determined with a 254 nm spectrophotometer. In addition, the distribution of mRNAs and proteins in each fraction was determined by qRT-PCR and western blot.

### Cycloheximide (CHX) treatment

Modified SNU-1 cells were seeded in 6-well plates and cultured with 5% CO2 at 37 °C. After 24 h, CHX was added to the wells at a final concentration of 100 μg/mL for 0 h, 4 h, 8 h, 12 h, 16 h, and 20 h. The cells at each time point were harvested, and western blotting was used to detect the protein expression of EGFR and mTOR.

### MG132 treatment

SNU-1 cells were seeded in 6-well plates and transfected into circIPO7-overexpressed plasmid and empty vector. After 48 h, MG132 was added to the wells at a final concentration of 25 μM for 24 h. The cells were harvested, and western blotting was used to detect the protein level of EGFR and mTOR.

### Statistical analysis

All of the statistical data were analyzed using GraphPad software, version 7.0 (GraphPad Prism, USA). The chi-square test was used to analyze the relationships between circRNA levels and clinicopathological factors in GC patients. Unpaired Student’s *t* test was used to analyze the differences between the two groups. The data are presented as the means ± SDs. *p* < 0.05 were considered to be significant.

## Supplementary information

Supplementary Figure S1

Supplementary Figure S2

Supplementary Figure S3

Supplementary Figure S4

Supplementary Figure S5

Supplementary Figure S6

Supplementary Figure Legend

Supplementary Table S1

Supplementary Table S2

Supplementary Table S3

Supplementary Table S4

Supplementary Table S5

## Data Availability

Expression analyses for protein in gastric cancer were obtained from the TCGA (https://portal.gdc.cancer.gov). The sequence analysis of circIPO7 (has_circ_0005092) was obtained from CircInteractome (https://circinteractome.nia.nih.gov/index.html) and circBank (http://www.circbank.cn/). Structure analysis of circIPO7 was downloaded from RNAfold (http://rna.tbi.univie.ac.at/cgi-bin/RNAWebSuite/RNAfold.cgi). Structure analysis of caprin-1 and its domains were obtained from UniProt (https://www.uniprot.org/) and Pfam (http://pfam.xfam.org/). Multiple sequence alignment was performed on EMBL Clustal Omega (https://www.ebi.ac.uk/Tools/msa/clustalo/). RNA-seq data are accessible at the GEO under accession number: GSE181769. Other data that support the findings of this study are available from the corresponding authors on reasonable request.
