# Supplementary figures and images for: circIPO7 dissociates caprin-1 from ribosomes and inhibits gastric cancer cell proliferation by suppressing EGFR and mTOR
Source: Oncogene. 2023 Feb 3;42(13):980–93. doi: 10.1038/s41388-023-02610-z (PMC10038803; doi:10.1038/s41388-023-02610-z)

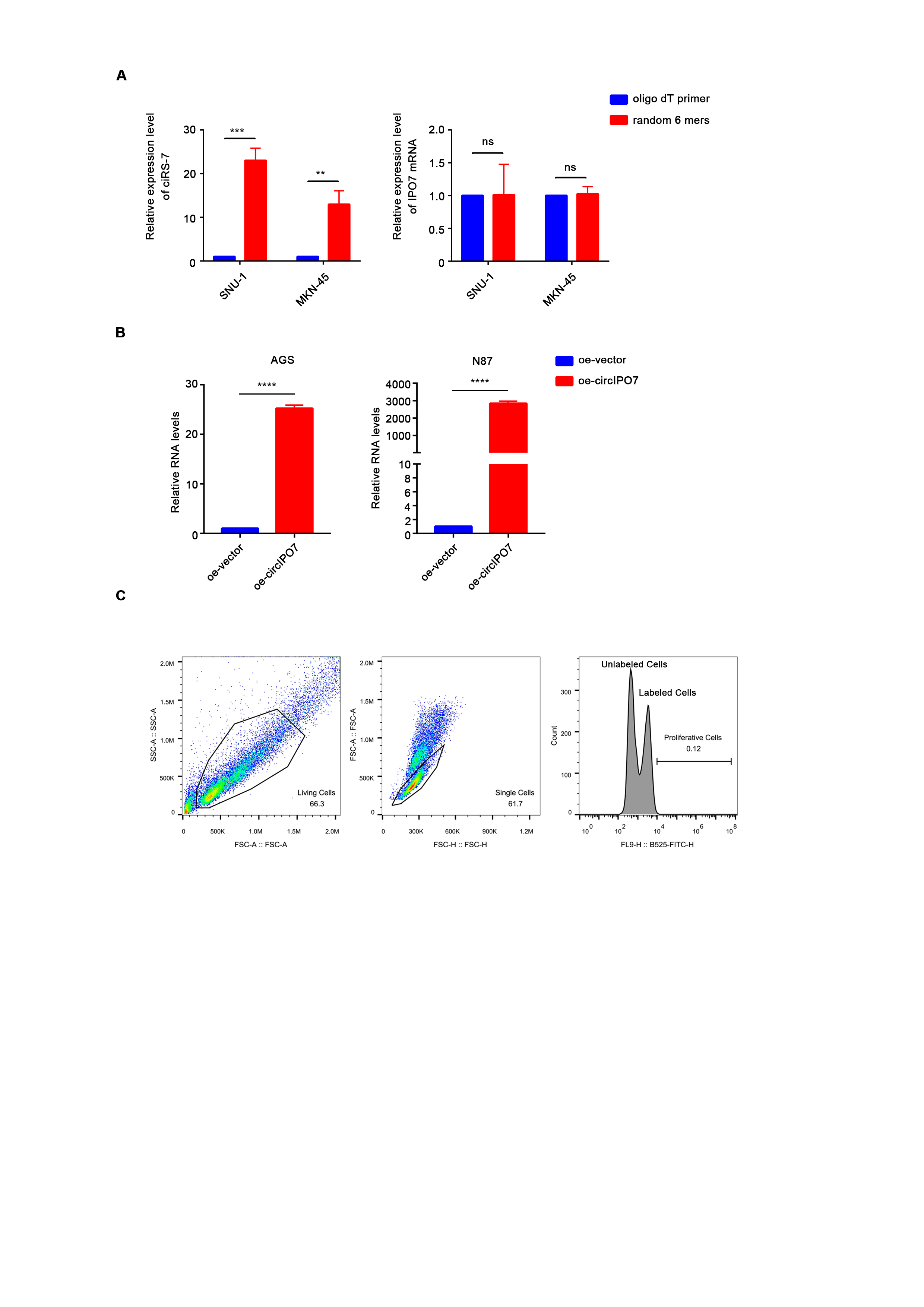

Supplement: Supplementary file 1 — Supplementary Figure S1 [file 41388_2023_2610_MOESM1_ESM.tif]

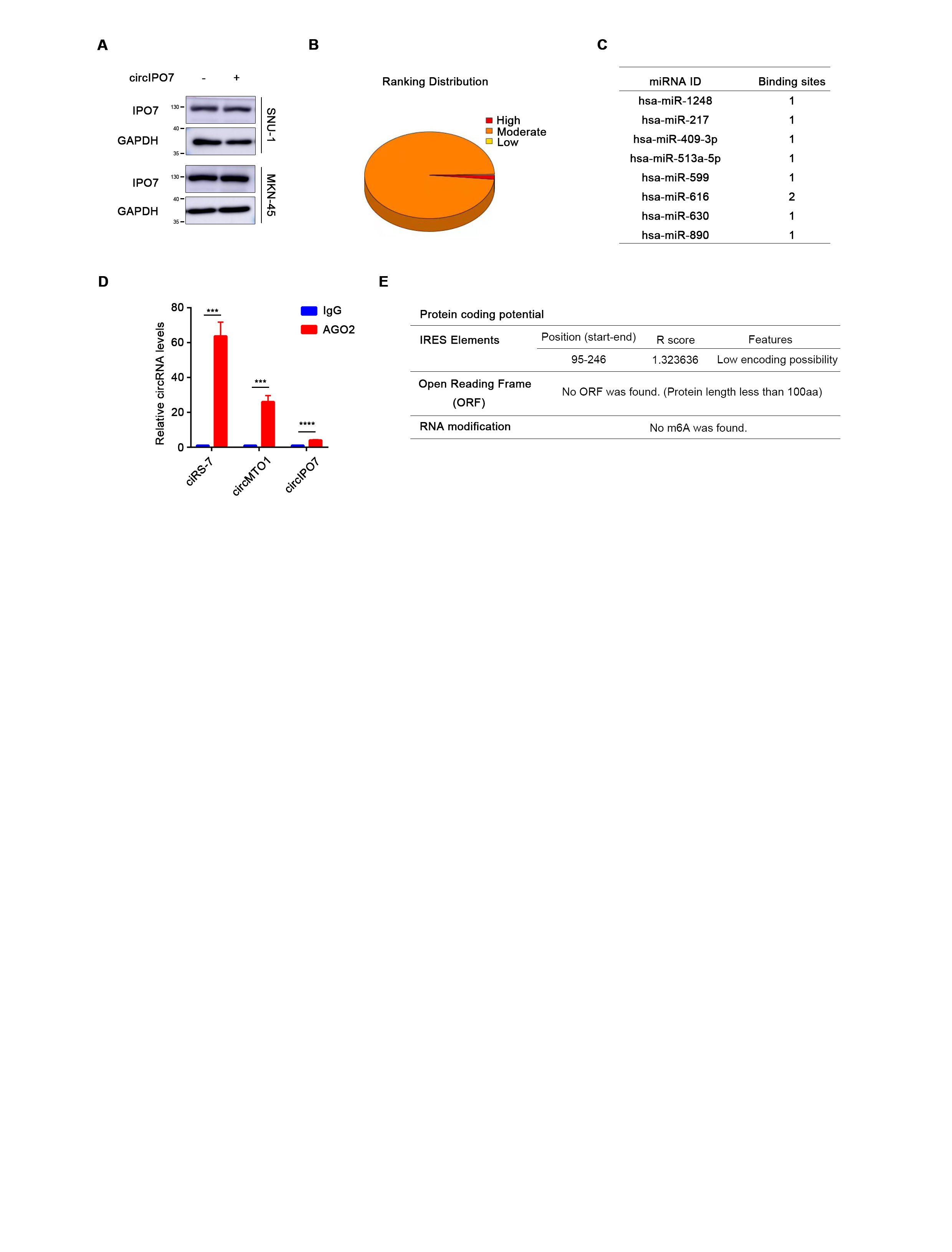

Supplement: Supplementary file 2 — Supplementary Figure S2 [file 41388_2023_2610_MOESM2_ESM.tif]

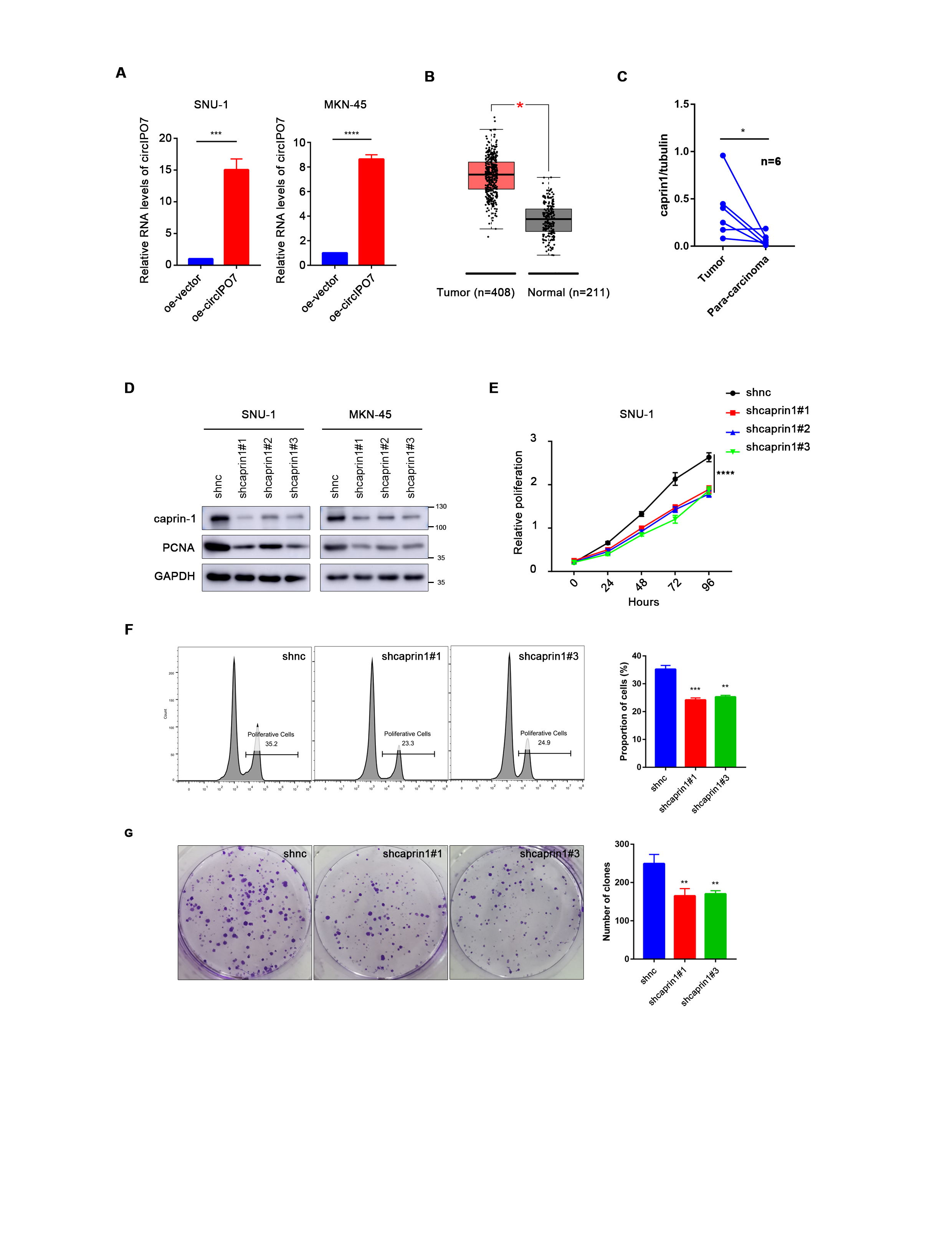

Supplement: Supplementary file 3 — Supplementary Figure S3 [file 41388_2023_2610_MOESM3_ESM.tif]

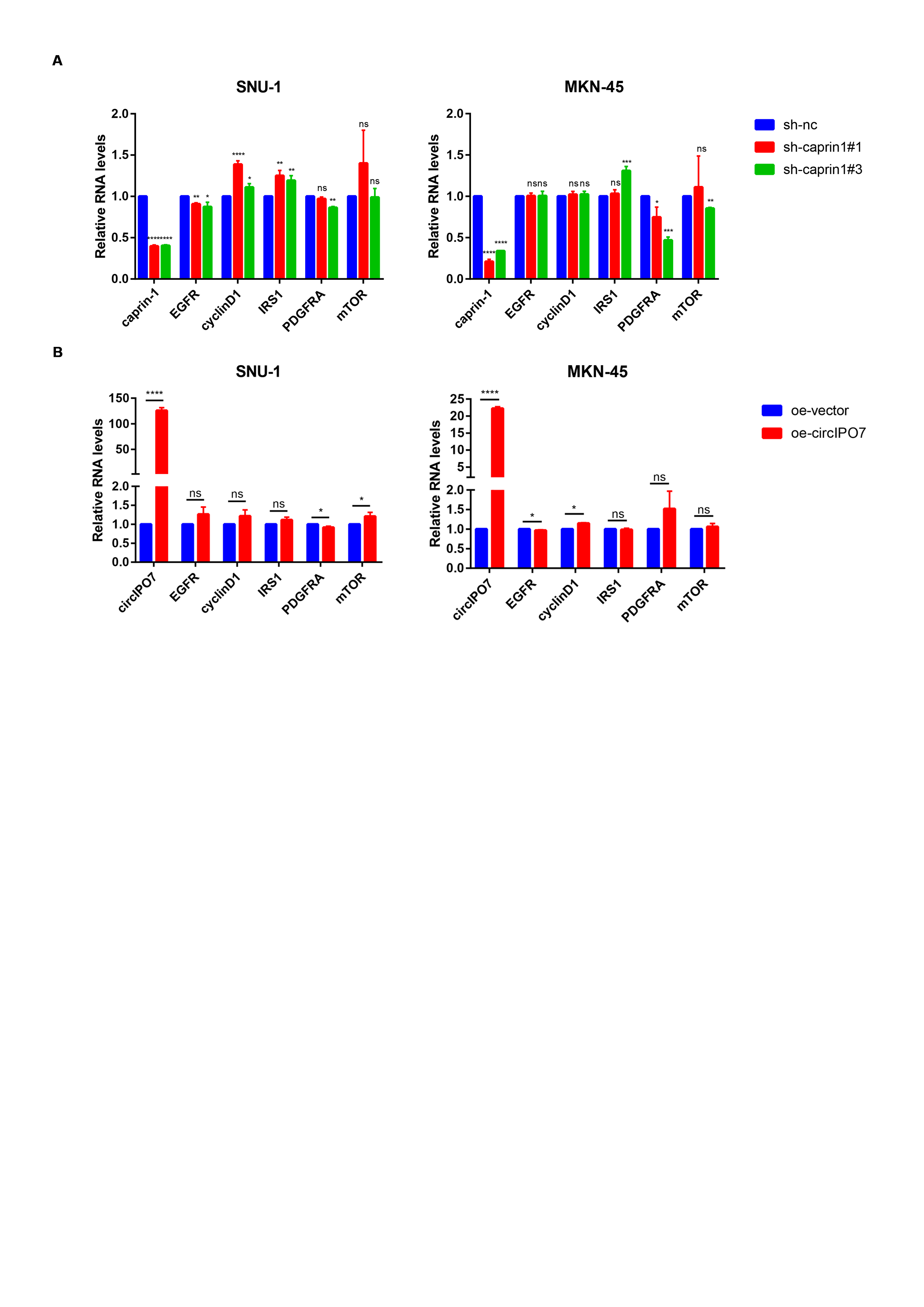

Supplement: Supplementary file 4 — Supplementary Figure S4 [file 41388_2023_2610_MOESM4_ESM.tif]

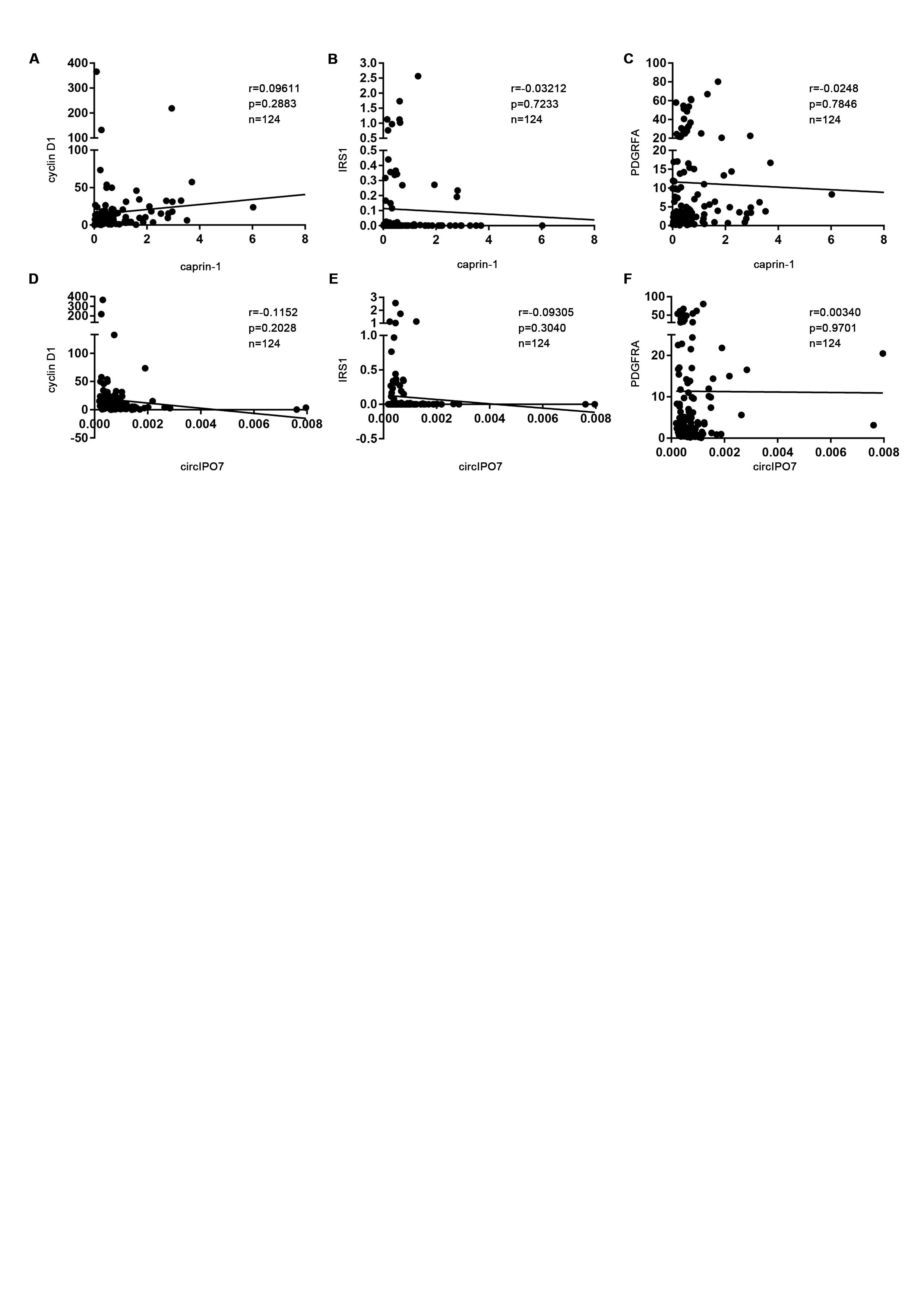

Supplement: Supplementary file 5 — Supplementary Figure S5 [file 41388_2023_2610_MOESM5_ESM.tif]

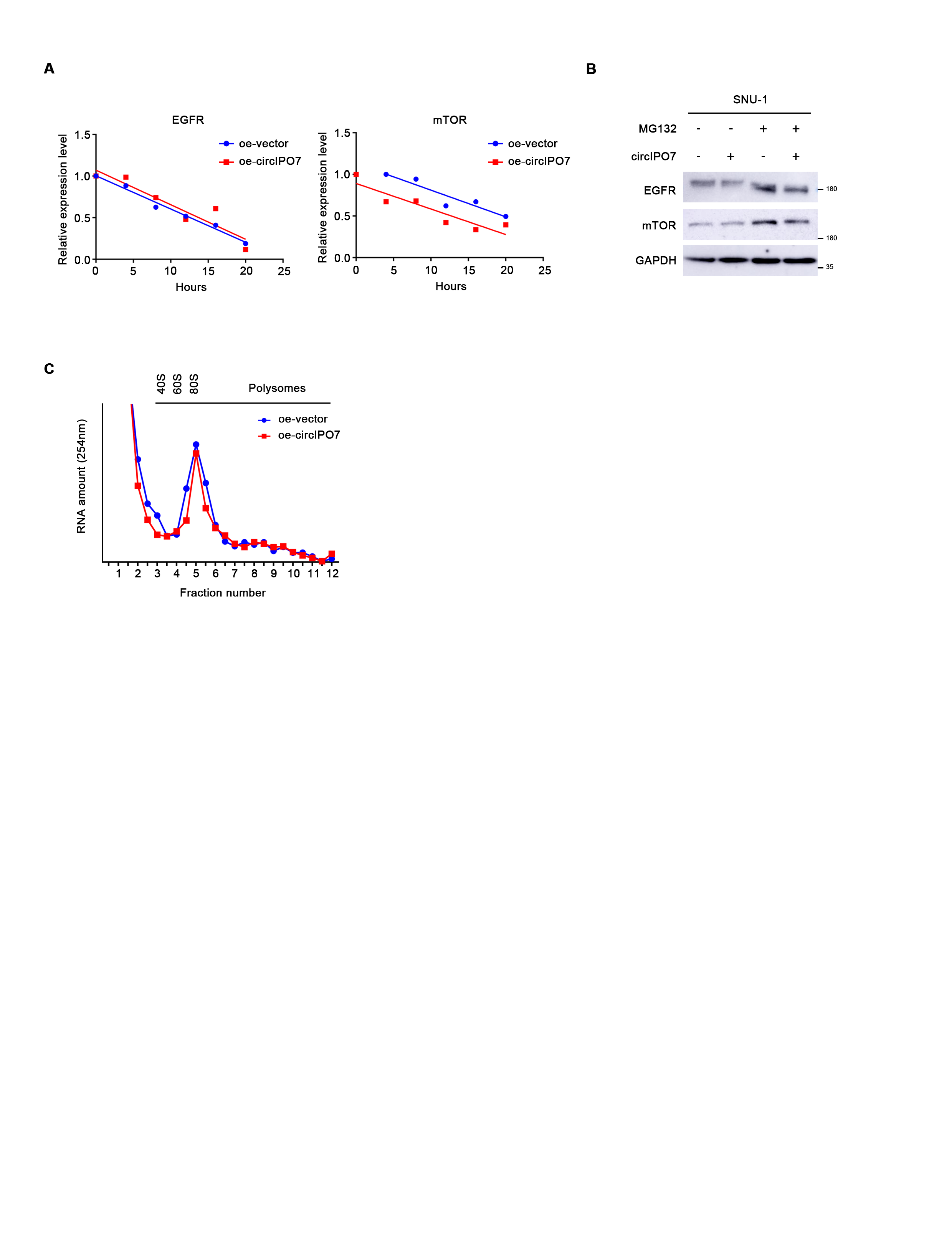

Supplement: Supplementary file 6 — Supplementary Figure S6 [file 41388_2023_2610_MOESM6_ESM.tif]
