# Supplementary material for: circIPO7 dissociates caprin-1 from ribosomes and inhibits gastric cancer cell proliferation by suppressing EGFR and mTOR
Source: Oncogene. 2023 Feb 3;42(13):980–93. doi: 10.1038/s41388-023-02610-z (PMC10038803; doi:10.1038/s41388-023-02610-z)
Supplement: Supplementary file 7 — Supplementary Figure Legend [file 41388_2023_2610_MOESM7_ESM.docx]

**Supplementary Figure**

**circIPO7 dissociates caprin-1 from ribosomes and inhibits gastric cancer cell proliferation by suppressing EGFR and mTOR**

Supplementary Figure 1. Identification of circIPO7 in GC

A. qRT-PCR analysis for ciRS-7 (typical circRNA with polyA- tail) and IPO7 mRNA (the host gene of circIPO7 with polyA+ tail) reverse-transcribed by random primers or oligo dT primers. ciRS-7, positive control. IPO7 mRNA, negative control.

B. qRT-PCR analysis was used to detect the overexpression efficiency of circIPO7 in AGS and N87 cells. oe, overexpression.

C. Screening strategy of the gating process for Edu assay. Cells that treated with Edu solution (10 μM) for 10 minutes were used as negative control. The two peaks from left to right are unlabeled cells and labeled but non-proliferative cells in the right image.

ns, no significant, **p<0.01, ***p<0.001, ****p<0.0001.

Supplementary Figure 2. Analysis of the potential mechanism of circIPO7

A. The expression of IPO7 protein in circIPO7-overexpressed cells was detected by western blot.

B. The protein binding potential of circIPO7 was predicted by catRAPID.

C. miRNA prediction of circIPO7 sequence via Circinteractome website.

D. RIP assay against AGO2 was preformed to reveal the potential of circIPO7 as miRNA sponge with the positive control of ciRS-7 and circMTO1.

E. Translation element prediction of circIPO7 sequence via circBank website.

****p*<0.001, *****p*<0.0001.

Supplementary Figure 3. The expression feature and biological function of caprin-1

A. RIP assay was used to verify the effect of circIPO7 overexpression on its interaction with caprin-1.

B. Caprin-1 expression in GC tissues (n=408) and paracarcinoma tissues (n=211) analyzed *via* TCGA website.

C. Caprin-1 expression in 6 paired GC tissues analyzed by western blot.

D. The expression of PCNA were examined in GC cells stably transfected with sh-caprin-1.

E-G. CCK-8 (E), Edu (F) and clone formation (G) analysis of the effect of caprin-1 knockdown on the GC cell proliferation.

**p*<0.05, ****p*<0.001, *****p*<0.0001.

Supplementary Figure 4. Effects of caprin-1 knockdown (A) and circIPO7 overexpression (B) on the mRNA levels of EGFR, cyclin D1, IRS1, PDGFRA and mTOR. ns, no significant, **p*<0.05, ***p*<0.01, ****p*<0.001, *****p*<0.0001.

Supplementary Figure 5. Pearson correlation analysis between the expression levels of cyclin D1, IRS1, PDGFRA with circIPO7 and caprin-1

A-C. Pearson correlation analysis between the expression levels of cyclin D1 (A), IRS1 (B), PDGFRA (C) with caprin-1 in 124 GC tissues.

D-F. Pearson correlation analysis between the expression levels of cyclin D1 (D), IRS1 (E), PDGFRA (F) with circIPO7 in 124 GC tissues.

Supplementary Figure 6. The pathway of circIPO7 affecting the protein level of EGFR and mTOR

A. CHX were used to inhibit protein synthesis in GC cells with vector- or circIPO7-overexpression, and subsequently, the protein degradation rate of EGFR and mTOR was analyzed.

B. GC cells with vector- or circIPO7-overexpression were treated with MG132 for 24 h, and then lysed to detect the protein levels of EGFR and mTOR by western blot.

C. Ribosomal profiling analysis of global RNA distribution in GC cells with vector- or circIPO7-overexpression.
